# Evaluating the effect of neighbourhood weight matrices on smoothing properties of Conditional Autoregressive (CAR) models

**DOI:** 10.1186/1476-072X-6-54

**Published:** 2007-11-29

**Authors:** Arul Earnest, Geoff Morgan, Kerrie Mengersen, Louise Ryan, Richard Summerhayes, John Beard

**Affiliations:** 1 Northern Rivers University Department of Rural Health, The University of Sydney, New South Wales, Australia; 2 Population Health & Planning, North Coast Area Health Service, New South Wales, Australia; 3 Faculty of Science, Queensland University of Technology, Queensland, Australia; 4 Department of Biostatistics, Harvard School of Public Health, Boston, USA; 5 Graduate Research College, Southern Cross University, New South Wales, Australia; 6 Centre for Urban Epidemiologic Studies. New York Academy of Medicine, New York, USA

## Abstract

**Background:**

The Conditional Autoregressive (CAR) model is widely used in many small-area ecological studies to analyse outcomes measured at an areal level. There has been little evaluation of the influence of different neighbourhood weight matrix structures on the amount of smoothing performed by the CAR model. We examined this issue in detail.

**Methods:**

We created several neighbourhood weight matrices and applied them to a large dataset of births and birth defects in New South Wales (NSW), Australia within 198 Statistical Local Areas. Between the years 1995–2003, there were 17,595 geocoded birth defects and 770,638 geocoded birth records with available data. Spatio-temporal models were developed with data from 1995–2000 and their fit evaluated within the following time period: 2001–2003.

**Results:**

We were able to create four adjacency-based weight matrices, seven distance-based weight matrices and one matrix based on similarity in terms of a key covariate (i.e. maternal age). In terms of agreement between observed and predicted relative risks, categorised in epidemiologically relevant groups, generally the distance-based matrices performed better than the adjacency-based neighbourhoods. In terms of recovering the underlying risk structure, the weight-7 model (smoothing by maternal-age 'Covariate model') was able to correctly classify 35/47 high-risk areas (sensitivity 74%) with a specificity of 47%, and the 'Gravity' model had sensitivity and specificity values of 74% and 39% respectively.

**Conclusion:**

We found considerable differences in the smoothing properties of the CAR model, depending on the type of neighbours specified. This in turn had an effect on the models' ability to recover the observed risk in an area. Prior to risk mapping or ecological modelling, an exploratory analysis of the neighbourhood weight matrix to guide the choice of a suitable weight matrix is recommended. Alternatively, the weight matrix can be chosen a priori based on decision-theoretic considerations including loss, cost and inferential aims.

## Background

The Conditional Autoregressive (CAR) model is widely used in small-area ecological studies to map outcomes measured at some areal level and to examine associations with covariates. Most of these applications are in the field of disease mapping (See Elliott for a list of studies [1]). The advantages of using the CAR model instead of presenting crude relative risks are well-described in the literature. One component of the CAR analysis is the use of a Bayesian model to include spatial association between observations. This approach offers a trade off between bias and variance reduction of the estimates, and has been shown to produce a set of point estimates that have improved properties in terms of minimising squared error loss, particularly in cases where the sample size is small [2].

When there is geographical correlation inherent in the data, ignoring such correlation can also lead to biased and inefficient inference, as the observations are strictly not independent. Elliott provides a summary of the various applications in disease mapping studies and some current methodological issues [1]. Lawson also highlights some of the applications of CAR models in disease mapping studies [3].

The CAR model was originally suggested by Besag [4] in the context of image analysis and is also known as the intrinsic CAR model with a convolution prior, or the Besag, York and Mollie (BYM) model. The original BYM model applied to continuous data that could be assumed to be normally distribution. In disease mapping studies, this has been adapted to incorporate normally distributed spatially correlated random effects into Poisson models for disease counts. The BYM model allows for the smoothing of relative risk estimate in each region towards the mean risk in the neighbouring areas. This provides for a more precise or reliable estimate of both mean and variance compared to using the crude rate. This is especially so, as the variance for the estimate of the raw rate with a small expected count can be large and unreliable. Risks are also smoothed towards the global mean to account for overdispersion. This 'shrinkage' of estimates towards the mean can be shown mathematically to be optimal if the aim is to minimise the squared-error loss, in a decision theory framework [5].

When undertaking CAR modelling of data at an areal level, it is necessary to define a, so-called, adjacency matrix that characterizes the neighbourhood structure of the data being analysed. There are several approaches to doing this, including defining neighbours according to the distances between centroids, declaring two regions to be neighbours if they share a boundary, and so on. It may also be necessary to specify the level of aggregation of data if small area data is available. Other influences include the choice of hyperprior distribution used for the precision estimates (e.g. gamma versus uniform), and the nature and sparseness of data. In this analysis, we are primarily concerned with the influence of different neighbourhood weight matrices on the amount of smoothing.

The CAR model has been used in many published studies, but only a few have provided justification on the choice of neighbourhood weight matrix structure. One exception is an ecological study to investigate the relationship between benzene emissions and the incidence of childhood leukaemia in Greater London, in which the authors considered three alternative levels of data aggregation in their analysis, and examined adjacency versus distance-based neighbourhood spatial weights for each of analysis [6]. For both the grid-level and ward-level analyses, they found that the adjacency based neighbourhood structure provided a better fit of the data, based on DIC (Deviance Information Criteria) comparisons. They also found a significant difference in the estimates of the spatially structured random effects between the distance-based and adjacency-based neighbourhood structures for the ward-level analysis for the model without any covariates. In another paper looking at the issue of spatial priors and single versus joint-disease models, the authors concluded that sensitivity to structural assumptions as well as hyperprior specification should be explored as part of any disease-mapping study [7].

A recent study of prostate cancer incidence in New York, recently published in the International Journal of Health Geographics, applied the CAR spatial model to obtain smoothed risk estimates at the ZIP-code level, and highlighted the use of distance-based weight functions in the model formulation [8]. While this paper included some sensitivity analysis on the choice of hyperpriors, the issue of neighbourhood weights was not addressed.

Other authors have used various specifications of the neighbourhood weight matrix. Wall [9] looked at spatial structure in the US SAT college entry exam results by state, having from one neighbour up to eight neighbours. A similar approached was used by Rasmussen [10] with Scottish lip cancer data and by MacNab [11] with chronic lung disease in neonatal intensive care units. Bell [12] used first-order neighbours for a spatial neighbour matrix in a CAR model describing the association of intrauterine growth restriction and area level covariates. English [13] mapped low birth weight to 0.5 km grids and used a CAR model to assess and adjust for residual spatial correlation using four neighbours for each cell (north, south, east and west) to describe spatial dependence. Kousa [14] geocoded acute myocardial infarctions in Finland to 10 km grids to examine spatial variation associated with geochemistry of ground water. The neighbours were defined using eight neighbours (side and corner) for each cell.

Abrial [15] used 23 km hexagonal grids to map cases of Bovine spongiform encephalopathy (BSE) Johnson [16] used adjacent zipcodes in a study on prostate cancer as the basis for geographical weighting. Other methods to define neighbours have included Euclidean distance [17], geographic distance and population size in prostate cancer [18].

In Australia, New South Wales (NSW) Health [19] recently used CAR models to produce smoothed maps of selected health indicators from 1999/2000 to 2003/2004 for 166 Local Government Areas in NSW. Similarly, the Cancer Council of NSW has provided on its website [20] maps of cancer incidence and mortality across New South Wales by Local Government Area (LGA) for the period 1998 to 2002. They used Bayesian methodology to smooth the maps of standardised incidence and mortality ratios.

The overall crude birth defect rate in NSW has decreased from 22.7/1,000 births in 1998 to 20.5 per 1,000 births in 2003 [21]. The only increases over this period were cases of chromosomal abnormalities which increased from 4.2 to 5.3, Cleft Palate (from 0.8 to 1.0 per 1000 births) and Down Syndrome which increased from 2.2 to 2.6 per 1,000 births. The spatial distribution of birth defects in NSW has only been published by very large spatial units (8 Area Health Services (AHS)) standardised for maternal age in the NSW population. The same report found that the Greater Southern AHS had the lowest rate of birth defects (15.7 per 1,000 births) and Hunter New England AHS had the highest (24.2 per 1,000 births) across the period 1998–2004. Spatial analysis at a smaller spatial unit than the 8 AHS within NSW will provide more information about the geographical distribution of the birth defects, and allow the development of hypotheses to explain this spatial variation.

More extensive assessment of the strengths and weaknesses of these, and other, possible approaches to neighbourhood weighting is urgently needed, since the results of analysis may vary substantially depending on the model chosen. Lawson [22] talks briefly about possible weighting schemes in the context of edge effects, including distance functions and surrogate measures derived from the perimeter of shared borders between neighbours. He also mentions the need to conduct sensitivity analysis on the choice of weights. However, no quantitative case-studies are shown to highlight this point. Best and colleagues [23], looked at the use of adjacency versus distance-based weights to define the neighbourhood structure for the residuals. However, their study examined just two specific weights (the rook adjacency and a distance decay function) and their results were based on a small dataset which included simulated risk structures. Model comparison was also done on the same data.

In another related study, Conlon [24] et al examined the effect of three different neighbourhood weight structures (namely fixed weights based on adjacency, parametric distance-based weights and distance-based covariances). For the limited data (again the Scottish lip cancer data) that they worked on, they found that the adjacency and variable covariance models seemed to provide better fit as compared to the variable distance model. In other words, they found differences according to the type of neighbours defined. Our study aims to look at a more comprehensive list of weight matrices, and the use of innovative measures to compare competing models.

## Aims

The main aims of our study were two-fold. Firstly, to explore any differences in the smoothing properties between the contiguity (adjacency) and distance-based methods of defining spatial weights. We also studied whether there were differences between the type and order of neighbours included within the contiguity method of neighbourhood definition. Secondly, for the distance-based method, we assessed the impact of including various formulations of the weight matrix. We performed external validation of the models by applying them to birth defects data in New South Wales.

## Methods

We obtained data on birth defects from the NSW Midwives Data Collection (MDC) and Birth Defects Register (BDR) databases from 1995 to 2003. We calculated standardised expected counts of total birth defects. For instance, the expected count of birth defects in each areal unit at a particular time period was defined as E_i _= (Births_i_/Totalbirths)*Totalbirthdefects, where Births_i _refers to total births in the _i_th SLA, and Totalbirths and Totalbirthdefects refer to the overall number of births and birth defects in the NSW study region for that particular time-period. Analyses were carried out at the SLA level, for which there were 198 SLAs defined within the NSW study area. These represent administrative districts that relate to local government jurisdictions. Statistical Local Area (SLA)-specific relative risk estimates were calculated as the ratio of the observed and expected counts for each area. Because a few of the SLA had no births recorded during a particular study period, we added a small constant (10^-5^) to both the numerator and denominator to ensure that the relative risks were well-defined. This constant is absorbed by the smoothing of these extreme relative risk estimates in the subsequent analysis. The data were grouped into 3 equal three-year long time periods: 1995–1997, 1998–2000 and 2001–2003. After confirming that these three time periods were similar with respect to relevant measures, we used the first two time-periods to build the model coefficients and then assessed model fit using data from 2001–2003. We excluded cases that had missing data for year of birth and maternal age. Year of birth was needed to assign the cases to each of the three time-periods, whereas maternal age was required to create one of the weight matrices. Also, to ensure that only good quality geocoded addresses was used, we excluded indeterminate geocodes, geocodes resulting in many addresses, many streets, many localities and those without any matches.

The New South Wales (NSW) Midwives Data Collection is a population-based surveillance system covering all births in NSW public and private hospitals, as well as home births. The information for each birth is recorded by either the attending midwife or medical practitioner. It encompasses all live births and stillbirths of at least 20 weeks gestation or at least 400 grams birth weight. The MDC receives notifications of women whose usual place of residence is outside NSW but who give birth in NSW. However, the MDC does not receive notifications of births outside NSW to women usually resident in NSW [21].

The New South Wales BDR is a population-based surveillance system established to monitor birth defects detected during pregnancy or at birth, or diagnosed in infants up to one year of age. The BDR was established in 1990 and, under the NSW Public Health Act 1991, from 1 January 1998 doctors, hospitals, and laboratories have been required to notify birth defects detected during pregnancy, at birth, or up to one year of life [21]. For the purposes of this statistical methodological study we considered all birth defects together.

The MDC and BDR data for 1990 to 2003 have recently been geocoded using software developed by NSW Health and the Australian National University and the geocoding process is described in detail elsewhere [25].

Ethical approval was obtained for the use of the NSW Midwives Data Collection and the NSW Births Defects Registry data from the NSW Department of Health Ethics Committee, and for the study itself from the University of Sydney Ethics Committee.

The formulation of the CAR model used in our analyses is shown below:

*O*_*ik *_~ *Poi*(μ_*ik*_)

log⁡(μ)ik=log⁡(Eik)+ui+vi+β1i∗tk+β2i∗tk2
 MathType@MTEF@5@5@+=feaafiart1ev1aaatCvAUfKttLearuWrP9MDH5MBPbIqV92AaeXatLxBI9gBaebbnrfifHhDYfgasaacPC6xNi=xI8qiVKYPFjYdHaVhbbf9v8qqaqFr0xc9vqFj0dXdbba91qpepeI8k8fiI+fsY=rqGqVepae9pg0db9vqaiVgFr0xfr=xfr=xc9adbaqaaeGacaGaaiaabeqaaeqabiWaaaGcbaGagiiBaWMaei4Ba8Maei4zaCMaeiikaGccciGae8hVd0MaeiykaKYaaSbaaSqaaiabdMgaPjabdUgaRbqabaGccqGH9aqpcyGGSbaBcqGGVbWBcqGGNbWzcqGGOaakcqWGfbqrdaWgaaWcbaGaemyAaKMaem4AaSgabeaakiabcMcaPiabgUcaRiabdwha1naaBaaaleaacqWGPbqAaeqaaOGaey4kaSIaemODay3aaSbaaSqaaiabdMgaPbqabaGccqGHRaWkcqWFYoGydaWgaaWcbaGaeGymaeJaemyAaKgabeaakiabgEHiQiabdsha0naaBaaaleaacqWGRbWAaeqaaOGaey4kaSIae8NSdi2aaSbaaSqaaiabikdaYiabdMgaPbqabaGccqGHxiIkcqWG0baDdaqhaaWcbaGaem4AaSgabaGaeGOmaidaaaaa@5C18@

where O_ik _and E_ik _are the observed and expected birth defects for a SLA in the *i*th region and *j*th time period, u_i _is a spatially structured random effect and v_i _is a spatially unstructured random effect. We also added a quadratic temporal random effect term to capture time trends. This model is an amended version from Bernardinelli [26]. The main difference lies in the exclusion of the space-time interaction random effect term; because the main focus of this paper is a comparison of the prior imposed on the spatial random effect, the inclusion of an interaction term would potentially blur this comparison and increase the computational cost substantially. Possible spatial correlation was accommodated in the model by introducing a conditional autoregressive (CAR) prior for the spatial random effects, as shown below (from Lawson AB 2003: Disease Mapping with WinBUGS and MLwiN).

[ui|uj,i≠j,τu2]~N(u¯i,τi2)
 MathType@MTEF@5@5@+=feaafiart1ev1aaatCvAUfKttLearuWrP9MDH5MBPbIqV92AaeXatLxBI9gBaebbnrfifHhDYfgasaacPC6xNi=xI8qiVKYPFjYdHaVhbbf9v8qqaqFr0xc9vqFj0dXdbba91qpepeI8k8fiI+fsY=rqGqVepae9pg0db9vqaiVgFr0xfr=xfr=xc9adbaqaaeGacaGaaiaabeqaaeqabiWaaaGcbaGaei4waSLaemyDau3aaSbaaSqaaiabdMgaPbqabaGccqGG8baFcqWG1bqDdaWgaaWcbaGaemOAaOMaeiilaWcabeaakiabdMgaPjabgcMi5kabdQgaQjabcYcaSGGaciab=r8a0naaDaaaleaacqWG1bqDaeaacqaIYaGmaaGccqGGDbqxcqGG+bGFcqWGobGtcqGGOaakcuWG1bqDgaqeamaaBaaaleaacqWGPbqAaeqaaOGaeiilaWIae8hXdq3aa0baaSqaaiabdMgaPbqaaiabikdaYaaakiabcMcaPaaa@4D6D@

u¯i=1∑jwij∑jujwij
 MathType@MTEF@5@5@+=feaafiart1ev1aaatCvAUfKttLearuWrP9MDH5MBPbIqV92AaeXatLxBI9gBaebbnrfifHhDYfgasaacPC6xNi=xI8qiVKYPFjYdHaVhbbf9v8qqaqFr0xc9vqFj0dXdbba91qpepeI8k8fiI+fsY=rqGqVepae9pg0db9vqaiVgFr0xfr=xfr=xc9adbaqaaeGacaGaaiaabeqaaeqabiWaaaGcbaGafmyDauNbaebadaWgaaWcbaGaemyAaKgabeaakiabg2da9KqbaoaalaaabaGaeGymaedabaWaaabuaeaacqWG3bWDdaWgaaqaaiabdMgaPjabdQgaQbqabaaabaGaemOAaOgabeGaeyyeIuoaaaGcdaaeqbqaaiabdwha1naaBaaaleaacqWGQbGAaeqaaOGaem4DaC3aaSbaaSqaaiabdMgaPjabdQgaQbqabaaabaGaemOAaOgabeqdcqGHris5aaaa@4469@

τi2=τu2∑jwij
 MathType@MTEF@5@5@+=feaafiart1ev1aaatCvAUfKttLearuWrP9MDH5MBPbIqV92AaeXatLxBI9gBaebbnrfifHhDYfgasaacPC6xNi=xI8qiVKYPFjYdHaVhbbf9v8qqaqFr0xc9vqFj0dXdbba91qpepeI8k8fiI+fsY=rqGqVepae9pg0db9vqaiVgFr0xfr=xfr=xc9adbaqaaeGacaGaaiaabeqaaeqabiWaaaGcbaacciGae8hXdq3aa0baaSqaaiabdMgaPbqaaiabikdaYaaakiabg2da9KqbaoaalaaabaGae8hXdq3aa0baaeaacqWG1bqDaeaacqaIYaGmaaaabaWaaabuaeaacqWG3bWDdaWgaaqaaiabdMgaPjabdQgaQbqabaaabaGaemOAaOgabeGaeyyeIuoaaaaaaa@3E14@

As we can see from the above equations, estimation of the risk in any area is conditional on risks in neighbouring areas. Subscripts i and j refer to an SLA and it's neighbour respectively, and j ε Ni where Ni represents the set of neighbours of region i. Besides the identification of neighbours, the assigned weights also affect the risk estimation. The weights for the adjacency and distance models are given by weights_ij _(w_ij_) = 1 if i,j are adjacent, and 0 otherwise. For the other distance-based models, various formulations of the weights (described in detail below) were used.

**Figure 1:**
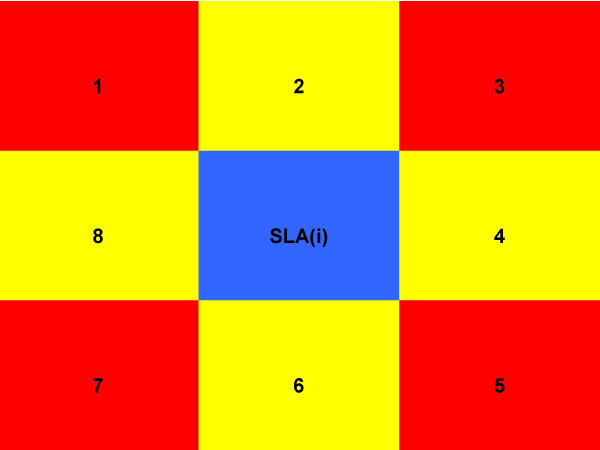
**Neighbourhood assignment based on adjacency**. Note: For Rook method, only neighbours 2,4,6 and 8 assigned to SLA(i). For Queen method, all neighbours (i.e. 1–8) are assigned to SLA(i).

We created four different neighbourhood adjacent weight matrices that are commonly used in spatial regression, namely Queen-1, Queen-2, Rook-1 and Rook-2. The numbers reflect the order of contiguity, and the main difference between the Queen and Rook method of assigning neighbours is that the latter uses only common boundaries to define neighbors, while the former includes all common points (boundaries and vertices). Please see Figure 1 for more details. For instance, the Queen-1 neighbourhood matrix for a SLA would include all its immediate neighbours that share common points with that area, while a Queen-2 matrix would include the immediate neighbours of the neighbours as well. All neighbours for the adjacency weight matrix contribute equal weights.

We also computed seven distance-based matrices. The simple distance-based matrix included all SLAs as neighbours and assigned them equal weights. Matrices Weight-1 to Weight-7 also include all SLAs as neighbours, but the weights were assigned differently. For Weights 1–3, the following formulations were used: w_ij _= 1/dist_ij_, w_ij _= 1/dist^2 ^_ij _and w_ij _= 1/dist^3 ^_ij _respectively. The weight matrix for the "Gravity" model was defined by w_ij _= e_i_e_j_/dist_ij_. The corresponding weight matrix for the "Entropy" model was defined by w_ij _= exp(-10*dist_ij_), and for the "Density" model: w_ij _= (1/dist_ij_)*density_i_*density_j_, with dist_ij _being the distance (decimal degrees) between the two SLAs, e_i _and e_j _being the standardised counts of births for an SLA and its respective neighbour, and density_i _and density_j _being the respective standardised birth population densities. A distance decay parameter of 10 was chosen, based on a preliminary exploratory examination of the correlogram of the relative risks over distance.

The weights for the "Gravity" and "Density" models were standardised against their mean and standard deviation for the purpose of comparability. Finally, for the Weight-7 model, the weights were w_ij _= 1/(dist_ij_*absolute(maternalage_i_-maternalage_j _+0.0001)), with maternalage_i _and maternalage_j _being the mean maternal ages in SLAs i and its neighbour j respectively. A small constant was needed to ensure that weights were defined for those pairs with identical values.

Our selection of the 7 distance-based models provides a variety of scenarios whereby the relative risks, in reality, are spatially correlated. The first three models (Weights 1–3) consider only distance in the weight function, placing greater weights on SLAs that are closer together. The "Gravity" model was used to examine whether placing greater weights on neighbours which themselves were relatively more populated, made a difference in smoothing. This was to examine the hypothesis that sparsely populated neighbours provide little information. The "Entropy" model was designed to provide a scenario whereby immediate neighbours were assigned most of the weights, and the weights were reduced drastically for those that were further away. This was done to exemplify a situation where one could expect localised environmental hazards to be present. The "Density" model was similar to the "Gravity" model, except that we weighted the neighbours according to the population density, instead of just the population.

For the covariate model, we chose maternal age mainly because this has been previously reported to be associated with birth defects in our study population (i.e. incidence of birth defects found to be increasing with maternal age in NSW [21]), and also because maternal age was less likely to be subject to recall bias as compared with other covariates, such as smoking.

The priors for the means were set to a normal distribution, with standard deviation set to cover a wide range of values, whereas the priors for the standard deviations of the precision estimates were set to a uniform distribution [27] with a wide yet plausible interval (i.e. range from 0.00001 to 20). This range was selected from initial exploratory analysis of the data.

We ran 12 different CAR models for the various adjacencies described above, using WinBUGS (version 1.4.1, Imperial College and Medical Research Council, UK). The models were run through Stata using a customised ado program file (written by Dr John Thompson, Department of Health Sciences, University of Leicester 2006). We discarded the first 40,000 samples as burn-in and ran a further 20,000 iterations which were used in the calculation of the posterior estimates. We ran two different chains, starting from diverse initial values and convergence was assessed using the Gelman-Rubin convergence statistic, as modified by Brooks and Gelman [28].

Estimates for the smoothed relative risk, posterior probability of relative risk greater than one, spatially structured random effect, spatially unstructured random effect and their corresponding 95% credible intervals were derived from the posterior distribution. We also computed the fraction of total random variation explained by the model as a ratio of the empirical variance of the spatial component against the total variance. This fraction provides us with a means to explore how much of the spatial variation in relative risk is explained by the model. The formulas are given below:

Var(u)=∑i(ui−u¯)2/(n−1)
 MathType@MTEF@5@5@+=feaafiart1ev1aaatCvAUfKttLearuWrP9MDH5MBPbIqV92AaeXatLxBI9gBaebbnrfifHhDYfgasaacPC6xNi=xI8qiVKYPFjYdHaVhbbf9v8qqaqFr0xc9vqFj0dXdbba91qpepeI8k8fiI+fsY=rqGqVepae9pg0db9vqaiVgFr0xfr=xfr=xc9adbaqaaeGacaGaaiaabeqaaeqabiWaaaGcbaGaemOvayLaemyyaeMaemOCaiNaeiikaGIaemyDauNaeiykaKIaeyypa0ZaaabeaeaacqGGOaakcqWG1bqDdaWgaaWcbaGaemyAaKgabeaakiabgkHiTiqbdwha1zaaraaaleaacqWGPbqAaeqaniabggHiLdGccqGGPaqkdaahaaWcbeqaaiabikdaYaaakiabc+caViabcIcaOiabd6gaUjabgkHiTiabigdaXiabcMcaPaaa@45B2@

Var(v)=∑i(vi−v¯)2/(n−1)
 MathType@MTEF@5@5@+=feaafiart1ev1aaatCvAUfKttLearuWrP9MDH5MBPbIqV92AaeXatLxBI9gBaebbnrfifHhDYfgasaacPC6xNi=xI8qiVKYPFjYdHaVhbbf9v8qqaqFr0xc9vqFj0dXdbba91qpepeI8k8fiI+fsY=rqGqVepae9pg0db9vqaiVgFr0xfr=xfr=xc9adbaqaaeGacaGaaiaabeqaaeqabiWaaaGcbaGaemOvayLaemyyaeMaemOCaiNaeiikaGIaemODayNaeiykaKIaeyypa0ZaaabeaeaacqGGOaakcqWG2bGDdaWgaaWcbaGaemyAaKgabeaakiabgkHiTiqbdAha2zaaraaaleaacqWGPbqAaeqaniabggHiLdGccqGGPaqkdaahaaWcbeqaaiabikdaYaaakiabc+caViabcIcaOiabd6gaUjabgkHiTiabigdaXiabcMcaPaaa@45B8@

*Fraction *= *Var*(*u*)/(*Var*(*u*) + *Var*(*v*))

u_i _is a spatially structured random effect and v_i _is a spatially unstructured random effect, with i ranging from 1 to n = 198.

We compared the different models in several ways. Firstly, we used the Deviance Information Criterion (DIC) developed by Spiegelhalter [29] to assess the complexity and fit of the models. The DIC is computed as the sum of the posterior mean deviance and estimated effective number of parameters.

*DIC *= *D *+ *p*_*D*_,

with *D *and p_D _being the sum of the posterior mean deviance and estimate of the effective number of parameters.

Generally, smaller values of DIC are preferred. We used the model-selection decision criteria suggested by Best [7], to suggest that models with DIC values within 1 or 2 of the 'best' model are also strongly supported, values within 3 and 7, weakly supported, and models with a DIC greater than 7 are substantially inferior.

For further model comparison, we also calculated the chi-squared residual sum of squares (RSS) to determine the amount by which the estimated counts of birth defects differed from the actual counts for the third time-period [3]. This is computed as the sum of the squared differences between the observed and estimated number of birth defects standardised by the estimated number of births:

RSS=∑(Oi−θ^i)2θ^i,
 MathType@MTEF@5@5@+=feaafiart1ev1aaatCvAUfKttLearuWrP9MDH5MBPbIqV92AaeXatLxBI9gBaebbnrfifHhDYfgasaacPC6xNi=xI8qiVKYPFjYdHaVhbbf9v8qqaqFr0xc9vqFj0dXdbba91qpepeI8k8fiI+fsY=rqGqVepae9pg0db9vqaiVgFr0xfr=xfr=xc9adbaqaaeGacaGaaiaabeqaaeqabiWaaaGcbaGaemOuaiLaem4uamLaem4uamLaeyypa0ZaaabqaKqbagaadaWcaaqaaiabcIcaOiabd+eapnaaBaaabaGaemyAaKgabeaacqGHsisliiGacuWF4oqCgaqcamaaBaaabaGaemyAaKgabeaacqGGPaqkdaahaaqabeaacqaIYaGmaaaabaGaf8hUdeNbaKaadaWgaaqaaiabdMgaPbqabaaaaaWcbeqab0GaeyyeIuoakiabcYcaSaaa@412A@

with O_i _and θ^i
 MathType@MTEF@5@5@+=feaafiart1ev1aaatCvAUfKttLearuWrP9MDH5MBPbIqV92AaeXatLxBI9gBaebbnrfifHhDYfgasaacPC6xNi=xI8qiVKYPFjYdHaVhbbf9v8qqaqFr0xc9vqFj0dXdbba91qpepeI8k8fiI+fsY=rqGqVepae9pg0db9vqaiVgFr0xfr=xfr=xc9adbaqaaeGacaGaaiaabeqaaeqabiWaaaGcbaacciGaf8hUdeNbaKaadaWgaaWcbaGaemyAaKgabeaaaaa@2F75@ being the observed and estimated number of birth defects respectively.

In order to determine the magnitude of smoothing in relation to epidemiologically meaningful cut-offs in the relative risks, we tabulated risk estimates into 3 groups, based on the 25^th ^and 75^th ^percentiles (Low: RR<0.65, Neutral: 0.65<RR<1.15, High: RR>1.15) and cross tabulated the observed with the predicted relative risk estimates. Using percentiles was a reasonable way to ensure enough numbers in each group to access sensitivity and specificity. To quantify the extent of change, we calculated the Kappa statistic [30], as a means to compare across models.

κ^=(po−pe)/(1−pe),
 MathType@MTEF@5@5@+=feaafiart1ev1aaatCvAUfKttLearuWrP9MDH5MBPbIqV92AaeXatLxBI9gBaebbnrfifHhDYfgasaacPC6xNi=xI8qiVKYPFjYdHaVhbbf9v8qqaqFr0xc9vqFj0dXdbba91qpepeI8k8fiI+fsY=rqGqVepae9pg0db9vqaiVgFr0xfr=xfr=xc9adbaqaaeGacaGaaiaabeqaaeqabiWaaaGcbaacciGaf8NUdSMbaKaacqGH9aqpcqGGOaakcqWGWbaCdaWgaaWcbaGaem4Ba8gabeaakiabgkHiTiabdchaWnaaBaaaleaacqWGLbqzaeqaaOGaeiykaKIaei4la8IaeiikaGIaeGymaeJaeyOeI0IaemiCaa3aaSbaaSqaaiabdwgaLbqabaGccqGGPaqkcqGGSaalaaa@3FCE@

with *p*_*o *_and *p*_*e *_being the observed and expected proportion of agreement respectively.

In addition, we computed and mapped the probability of a relative risk (RR) more than one. Although a cut-off of 0.7 has been shown to provide reasonable sensitivity to detect areas with an elevated risk [31] for a range of scenarios having moderate expected counts and excess risks of about 1.5, we estimated our own optimal cut-offs using the receiver-operating characteristic (ROC) curve.

Data extraction, management, analysis and diagnostics were done in Stata (version 9.2, Stata Corp, College Station, USA) and the maps were produced in Stata as well as ArcMap version 9.0 (ESRI, USA). All the weight matrices were created using the GeoDA software (version 0.9.5-I, University of Illinois, USA) and Stata.

## Results

The number of birth defects recorded during the time periods 1995–1997, 1998–2000 and 2001–2003 that we used in the analysis were 5924, 6161 and 5510 respectively. The total number of births during the same study-periods were 257353, 258147 and 255138 respectively. The mean number of first-order neighbours is shown in Table 1. For the Queen method of assignment, a mean of 5 neighbours were identified; for second-order assignment, the mean number of neighbours increased by about three-fold. There was little difference in the number of neighbours assigned by the Queen and Rook method due to the irregularity of the SLA areal units. The distance-based method of assignment resulted in all SLAs having 197 neighbours (i.e. all areas were considered neighbours).

**Table 1:** Characteristics of neighbourhood weight matrices

**Neighbourhood Type**	**Mean**	**Median**	**Min**	**Max**	**SD**	**Sum**
Queen-1	5	5	1	12	2	960
Queen-2	15	15	3	29	6	3018
Rook-1	5	5	1	11	2	956
Rook-2	15	15	3	29	6	3010
Distance	197	197	197	197	NA	39006

Next, we compared the characteristics of the neighbourhood types in terms of various measures (Table 2). Generally, there was greater agreement between observed and predicted relative risks, categorised in quartiles, using the distance-based matrices compared to the adjacency-based neighbourhoods. The Queen-1 model had a low Kappa value of 0.05, indicating a larger amount of smoothing. Among the distance-based models, the 'Gravity' model (Weight-4) performed better with a Kappa of 0.15. The 'Covariate' model fell in between the adjacency and distance-based models.

**Table 2:** Comparison of model fit and sensitivity of detecting areas with an elevated risk

**Neighbourhood type**	**Kappa**	**Fraction**	**DIC**	AUC*	**Sensitivity of detecting SLAs with elevated risks (PP cut-off = 0.33)**	**Specificity**
Queen-1	0.05	22%	2283	0.61	77%	41%
Queen-2	0.05	51%	2282	0.62	78%	43%
Rook-1	0.05	14%	2282	0.62	76%	41%
Rook-2	0.05	41%	2282	0.62	78%	39%
Distance	0.07	41%	2274	0.62	80%	30%
Weight-1 (1/distance)	0.07	41%	2213	0.62	80%	30%
Weight-2 (1/distance^2^)	0.12	98%	2204	0.61	75%	44%
Weight-3 (1/distance^3^)	0.08	93%	2218	0.59	75%	46%
Weight 4 (Gravity)	0.15	99%	2202	0.60	74%	39%
Weight 5 (Entropy)	0.08	91%	2227	0.60	74%	47%
Weight 6 (Density)	0.14	97%	2208	0.60	74%	37%
Weight 7 (Covariate)	0.10	95%	2210	0.62	74%	47%

* Area under curve from ROC analysis

In terms of fraction of random effect due to 'spatially structured random effects', the adjacency-based models, generally, had lower values as compared with the distance-based models. For instance, when we used the Queen-1 matrix, only 22% of the variation in the relative risks could be attributed to spatial effects. Among the distance-based models, the 'Gravity' model had the highest value (fraction = 99%), followed by Weight-2 (fraction = 98%) and 'Density' models (fraction = 97%).

When we examined the DIC as a basis of model-selection, we found that the 'Gravity' model had the lowest DIC of 2202, followed by Weight-2 (DIC = 2204) and 'Density' model (DIC = 2208). Generally, the adjacency-based matrices had higher DICs.

Using the decision tool suggested by Richardson *et al.*[31], we also compared the models in terms of their ability to detect areas with an elevated risk (i.e. RR>1.15). When we used the cut-off of 0.7 as suggested by their study, we found that all the models performed well in terms of specificity, but poorly in terms of sensitivity (data not shown).

The ROC analysis indicated that the posterior probabilities from the distance-based models had a similar discriminatory ability (i.e. same area under the curve) compared to the adjacency-based models (Table 2). However, the sensitivities can be improved by choosing a different threshold. When we used a threshold of 0.33 as determined by our ROC analysis to optimise sensitivity, we found some improvements in the models. For instance, the Queen-1 model was now able to correctly classify 36/47 high-risk areas (sensitivity 77%) with a specificity of 44%. The distance-based neighbourhood matrices had similar ranges of sensitivities and specificities. As with all such models, the choice of threshold depends on the inferential aims of the analysis, including the loss or cost associated with making a wrong positive or negative decision.

Finally, we compared the amount of smoothing performed by the various models. The distance-based models generally had lower RSS values as compared with adjacency-based models, indicating a lower amount of smoothing, and hence a better ability to predict the observed risk in an area. Most of the smoothing occurred in areas with low expected counts (Table 3) as expected.

**Table 3:** Comparison of amount of smoothing performed, stratified by size of population

**Neighbourhood type**	**RSS**	RSS (Areas with low expected count)*	**RSS (Areas with high expected count)**
Queen-1	91427	91254	173
Queen-2	91349	91176	174
Rook-1	91150	90976	174
Rook-2	90975	90802	173
Distance	88987	88816	171
Weight-1 (1/distance)	88987	88816	171
Weight-2 (1/distance^2^)	89612	89436	176
Weight-3 (1/distance^3^)	92666	92490	176
Weight 4 (Gravity)	87510	87330	179
Weight 5 (Entropy)	90550	90378	172
Weight 6 (Density)	87336	87162	174
Weight 7 (Covariate)	79478	79292	185

* Expected count less than median value of 9

**Figure 2:**
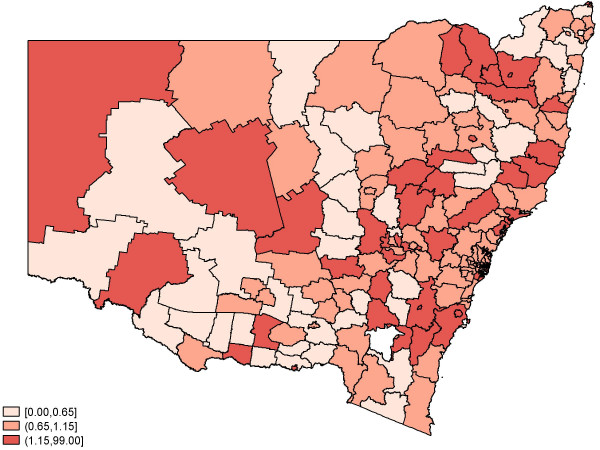
Observed relative risk of birth defects: 2001–2003.

**Figure 3:**
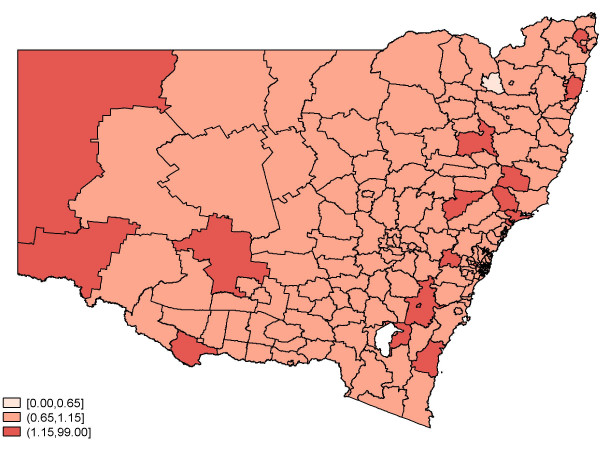
**Predicted relative risk of birth defects: 2001–2003**. Queen -1 Model.

**Figure 4:**
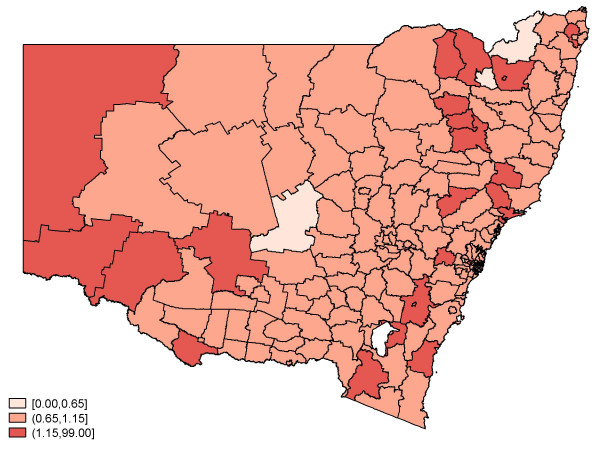
**Predicted relative risk of birth defects: 2001–2003**. Weights- 7 Covariate Model.

Figure 2 depicts the crude (observed) standardised relative risk of birth defects by SLA regions. There appear to be pockets of areas with an elevated risk, and these areas seem to be surrounded by regions with similar risk values. Figures 3 and 4 feature the predicted relative risk of birth defects using the Queen-1 adjacency neighbourhood matrix and the 'Covariate' method of assigning weights. It is apparent that the latter seems to perform better in recovering the true underlying relative risk.

In terms of diagnostics, the Gelman and Rubin plots indicated convergence after about 20,000 iterations for the posterior estimates of the regression coefficients, 3 randomly selected relative risk estimates and 3 randomly selected posterior probability estimates. The 'fraction' parameters took a longer time to converge (around 40,000 iterations); thus for consistency, we discarded the first 40,000 iterations for all the parameters. Due to the complex weight structures, the models were computationally intensive, and a further 20,000 iterations provided us with a reasonable Monte Carlo standard error (less than 1% of the standard deviation) for the estimates.

## Discussion

We found considerable differences in the smoothing properties of the CAR model, depending on the type of neighbours specified. This in turn had an effect on the models' ability to predict the observed risk in an area. These results have significant implications for all researchers using CAR models, since the neighbourhood weight matrices chosen may markedly influence a study's findings.

For instance, if one were primarily concerned with classifying areas into low/high risk of birth defects, the distance-based models appear to perform better than the adjacency-based ones. These models also have a higher Kappa, indicating low levels of changes in relative risk estimates across epidemiologically relevant thresholds. While we used Kappa with equal weights for the different categories, it is possible to use a weighted Kappa instead and assign higher weights for more important categories (e.g. smoothing a high relative risk).

If the aim were to explain away the spatial relationship in the relative risk estimates, then again distance-based models, such as 'Gravity' or Weight-2 models might be useful. Conversely, if one wants to preserve the spatial structure of the relative risks and examine the relationship between covariates that were spatial in nature, then one might prefer a model that has a low fraction of random effect due to spatially structured random effects to begin with.

The DIC appears to reflect the choice of models based on the level and nature of smoothing performed. For instance, the model with the lowest DIC, 'Gravity', had a high fraction (99%) and a high Kappa (0.15) as well. The Weight-2 model also seems to be 'strongly supported'. However, reliance on just the DIC alone fails to account for the nature of spatial relationship inherent in the model.

In terms of detecting areas with an elevated risk, we found that using information from the posterior probability had a higher sensitivity than looking at the smoothed relative risks alone. For instance, in the 'Covariate' model, using the smoothed (predicted) relative risk allowed us to detect 11 (22%) of the 50 SLAs with an elevated risk, as compared to 74% if we used information from the posterior probability instead.

As illustrated by the ROC analysis, the choice of threshold in the posterior probabilities involves a trade-off between sensitivity and specificity. Depending on the aims of the study, one should choose an appropriate cut-off point, using the ROC analysis. If for instance, one were to undertake exploratory spatial analysis to generate hypothesis on possible explanatory factors of elevated birth defect rates at an areal level, one could choose a low sensitivity but with a high specificity, so that the false negatives are minimised.

Our finding that distance-based neighbourhood models perform better than adjacency models is not surprising. This can be attributed to the highly irregular shapes and sizes of the SLAs (see Figure 1). The adjacency models can be expected to perform better with regular shaped areas such as grids.

The Kappa values in our study ranged from 0.05 to 0.14. While these may seem to afford modest values of agreement, we do note that these were ecological models and the models were evaluated on an 'external' time-period.

It is also worth mentioning the relatively good performance of the 'Gravity' model, which had the highest Kappa, the highest fraction of random effect due to spatially structured random effects and the lowest DIC. One current limitation of the CAR model is that it 'borrows strength' from neighbours even if they themselves are sparsely populated. The 'Gravity' model weighs neighbours according to the size of their population, and we have shown that this improves precision. The importance will be more marked for sparsely populated maps.

In our analysis, we used the simple spatial formulation of the CAR model comprising of spatially structured and spatially unstructured random effects and a temporal term. The purpose was not to make comparisons with the other formulations of the CAR model (e.g. mixture model, spatio-temporal interaction models, multivariate CAR models, etc), but further research might examine if these results reported here can be replicated in those models as well. This is also not the first time this particular space-time model has been applied to study epidemiological data. Assuncao and colleagues have used a similar model to map and project the rates of visceral Leishmaniasis in Belo Horizonte, Brazil across 117 study areas and 3 time points [32]. For our CAR models, we also used the Uniform priors on the standard deviations, instead of a Gamma prior on the precision estimates, as recent research has identified problems associated with the Inverse-Gamma prior [27], in particular the poor performance of the prior in terms of being non-informative.

The thresholds used in our definition of epidemiologically relevant cut-offs for the relative risks in our study may seem arbitrarily selected (based on the 25^th ^and 75^th ^percentiles). However, the same thresholds were used for all models, thus ensuring comparability, and we do not believe that the choice of alternative cut-offs would affect inferences.

It was also not the aim of this paper to undertake studies of association or ecological regression models, although covariates and interactions can easily be included in the existing models. Our research group is currently undertaking work to examine the impact of socio-economic status, demographic and environmental risk factors of adverse birth outcomes at an areal-level. A complete understanding of the structural form of CAR models is needed before this process of ecological modelling is pursued. Future work on incorporating landscape features and spatial smoothing based on additional covariates (e.g. multivariate similarity index) may improve the performance of the CAR models in disease mapping studies.

## Conclusion

Disease mapping studies that make use of the CAR model to smooth relative risks at some areal level need to take into account structural forms of the model specified. Depending on the aims of the study, various forms of the neighbourhood weight matrices provide differential levels of smoothing. Prior to risk mapping or ecological modelling, the weight matrix should be chosen according to the inferential and decision-theoretic aims of the study or through an exploratory analysis of the nature and degree of spatial correlation. In addition, a sensitivity analysis on the choice of neighbourhood weight matrix should be performed.

## Competing interests

The author(s) declare that they have no competing interests.

## Authors' contributions

AE participated in the design of the study, performed the statistical analysis and the draft of the manuscript. GM and RS contributed to the geo-coding process and helped to draft the manuscript. KM, LR and JB participated in the study design and helped to draft the manuscript. All authors read and approved the final manuscript.

## References

[B1] Elliott P, Wartenberg D (2004). Spatial epidemiology: current approaches and future challenges. Environmental Health Perspectives.

[B2] Carlin BP, Louis TA (2000). Bayes and empirical Bayes methods for data analysis.

[B3] Lawson AB, Biggeri AB, Boehning D, Lesaffre E, Viel JF, Clark A, Schlattmann P, Divino F (2000). Disease mapping models: an empirical evaluation. Disease Mapping Collaborative Group. Stat Med.

[B4] Besag J, York J, Mollie A (1991). Bayesian image restoration with application in spatial statistics. Ann Inst Math Stat.

[B5] Gelman A, Carlin JB, Stren HS, Rubin DB (2003). Bayesian Data Analysis.

[B6] Best N, Cockings S, Bennett J, Wakefield J, Elliott P (2001). Ecological regression analysis of environmental benzene exposure and childhood leukaemia: sensitivity to data inaccuracies, geographical scale and ecological bias. J R Statist Soc A.

[B7] Best N, Richardson S, Thomson A (2005). A comparison of Bayesian spatial models for disease mapping. Stat Methods Med Res.

[B8] Johnson GD (2004). Small area mapping of prostate cancer incidence in New York State (USA) using fully Bayesian hierarchical modelling. Int J Health Geogr.

[B9] Wall MM (2004). A close look at the spatial structure implied by the CAR and SAR models. Journal of Statistical Planning and Inference.

[B10] Rasmussen S (2004). Modelling of discrete spatial variation in epidemiology with SAS using GLIMMIX. Computer Methods & Programs in Biomedicine.

[B11] MacNab YC (2003). Hierarchical Bayesian spatial modelling of small-area rates of non-rare disease. Statistics in Medicine.

[B12] Bell BS, Beam C (2002). Spatial analysis of disease – applications. Biostatistical Applications in Cancer Research.

[B13] English PB, Kharrazi M, Davies S, Scalf R, Waller L, Neutra R (2003). Changes in the spatial pattern of low birth weight in a southern California county: the role of indivdual and neighbourhood level factors. Social Science & Medicine.

[B14] Kousa A, Moltchanova E, Viik-Kajander M, Rytkonen M, Tuomilehto J, Tarvainen T, Karvonen M (2004). Geochemistry of ground water and the incidence of acute myocardial infarction in Finland. J Epidemiol Community Health.

[B15] Abrial D, Calavas D, Jarrige N, Ducrot C (2005). Spatial heterogeneity of the risk of BSE in France following the ban of meat and bone meal in cattle feed. Preventive Veterinary Medicine.

[B16] Johnson GD (2004). Small area mapping of prostate cancer incidence in New York State (USA) using fully Bayesian hierarchical modelling. International Journal of Health Geographics.

[B17] Cressie N, Chan NH (1989). Spatial modelling of regional variables. Journal of the American Statistical Association.

[B18] Lopez FJA, Sanmartin P, Lawson AB, et al (1999). Spatial regression models in epidemiological studies. Disease mapping and risk assessment in public health.

[B19] Public Health Division The health of the people of New South Wales – Report of the Chief Health Officer. Sydney. NSW Department of Health.

[B20] Bois JP, Clements MS, Yu XQ, Supramaniam R, Smith DP, Bovaird S, O'Connell DL (2007). Cancer Maps for New South Wales 1998 to 2002.

[B21] Centre for Epidemiology and Research, NSW Department of Health (2005). New South Wales Mothers and Babies 2004. N S W Public Health Bull.

[B22] Lawson AB (2006). Statistical Methods in Spatial Epidemiology.

[B23] Best NG, Arnold RA, Thomas A, Waller LA, Conlon EM, Bernardo JM, Berger JO, David AP, Smith (1999). Bayesian models for spatially correlated disease and exposure data (with discussion). In Proceedings of the 6th Valencia International Meetings on Bayesian Statistic Bayesian Statistics 6.

[B24] Conlon EM, Waller LA (1999). Flexible spatial hierarchical models for mapping disease rates. In Proceedings of ASA Section on Statistics and the Environment: Washington, DC.

[B25] Summerhayes R, Holder P, Beard J, Morgan G, Christen P, Willmore A, Churches T (2006). Automated geocoding of routinely collected health data in New South Wales. N S W Public Health Bull.

[B26] Bernardinelli L, Clayton D, Pascutto C, Montomoli C, Ghislandi M, Songini M (1995). Bayesian analysis of space-time variation in disease risk. Statistics in Medicine.

[B27] Gelman A (2005). Prior distributions for variance parameters in hierarchical models. Bayesian Analysis.

[B28] Brooks SP, Gelman A (1998). Alternative methods for monitoring convergence of iterative simulations. Journal of Computational and Graphical Statistics.

[B29] Spiegelhalter DJ, Best NG, Carlin BP, Van der Linde A (2002). Bayesian measures of model complexity and fit (with discussion). J Roy Statist Soc B.

[B30] Cohen J (1960). A coefficient of agreement for nominal scales. Educational and Psychological Measurement.

[B31] Richardson S, Thomson A, Best N, Elliott P (2004). Interpreting posterior relative risk estimates in disease-mapping studies. Environ Health Perspect.

[B32] Assunção RM, Reis IA, Oliveira CD (2001). Diffusion and prediction of Leishmaniasis in a large metropolitan area in Brazil with a Bayesian space-time model. Stat Med.

